# A Brief Analysis of Suicide Methods and Trends in Virginia from 2003 to 2012

**DOI:** 10.1155/2015/104036

**Published:** 2015-02-01

**Authors:** Sameer Hassamal, Lori Keyser-Marcus, Ericka Crouse Breden, Kathrin Hobron, Atit Bhattachan, Ananda Pandurangi

**Affiliations:** ^1^Department of Psychiatry, Virginia Commonwealth University, P.O. Box 980710, Richmond, VA 23298, USA; ^2^Office of the Chief Medical Examiner, Department of Forensic Epidemiology, Virginia Department of Health, P.O. Box 2448, Richmond, VA 23219, USA; ^3^Department of Psychiatry, University of Pittsburgh, P.O. Box 850, Pittsburgh, PA 15213, USA

## Abstract

*Background.* The objective is to analyze and compare Virginia suicide data from 2003 to 2012 to US suicide data.* Methods.* Suicide trends by method, age, gender, and race were obtained from Virginia's Office of the Chief Medical Examiner's annual reports.* Results.* Similar to US suicide rates, suicide rates in Virginia increased between 2003 and 2012 from 10.9/100,000 people to 12.9/100,000 people. The most common methods were firearm, asphyxia, and intentional drug overdose, respectively. The increase in asphyxia (*r* = 0.77, *P* ≤ 0.01) and decrease in CO poisoning (*r* = −0.89, *P* ≤ 0.01) were significant. Unlike national trends, intentional drug overdoses decreased (*r* = −0.55, *P* = 0.10). Handgun suicides increased (*r* = 0.61, *P* = 0.06) and are the most common method of firearm suicide. Hanging was the most common method of asphyxia. Helium suicides also increased (*r* = 0.75, *P* = 0.05). Middle age females and males comprise the largest percentage of suicide. Unlike national data, the increase in middle age male suicides occurred only in the 55–64-year-old age group (*r* = 0.79, *P* ≤ 0.01) and decreased in the 35–44-year-old age group (*r* = −0.60, *P* = 0.07) and 10–14-year-old age group (*r* = −0.73, *P* = 0.02). Suicide in all female age ranges remained stable. Caucasians represent the highest percentage of suicide.* Conclusion. *There has been a rise in suicide in Virginia and suicide rates and trends have closely resembled the national average albeit some differences. Suicide prevention needs to be enhanced.

## 1. Introduction

Suicide is a self-inflicted destructive attempt to end one's life, which has multiple causes that are divided into proximal stressors and predisposition [1]. An acute exacerbation of an underlying psychiatric diagnosis is the primary stressor for suicide; however other stressors could include a psychosocial crisis [1]. Although, it is difficult to predict who will attempt suicide, a number of factors may increase the risk for suicide, including gender, support system, genetic liability, childhood experiences, and the availability of lethal means [1]. Individuals at a greater risk for completed suicide have also been found to be male, older, impulsive, and to have multiple physical ailments, a history of prior suicide attempts, a history of psychiatric illness, a history of violence, and a family history of suicide [2, 3].

The incidence of suicide worldwide is staggering with one million suicides annually [4]. The suicide method varies from one region to the next, although hanging is a common method used across all cultures and regions [5, 6]. Between 1992 and 2002 in the US, hanging accounted for 20.4% of suicides in men and 16.9% of suicides in females [6]. Firearm was the most common method of suicide in Argentina, Switzerland, Uruguay, and the United States. From 1992 to 2002, firearm use constituted 60.6% of male suicides and 35.7% of female suicides in the US [5, 6]. Pesticide suicides have constituted a large proportion of suicides in rural Latin American countries and Asian countries [5]. In contrast, from 1992 to 2002, pesticide suicide constituted only 0.3% of male suicides and 0.5% of female suicides in the US [5, 6]. Although intentional drug poisoning is common in Canada, the US, and Northern Europe, it does not constitute the majority of suicides [5, 6]. From 1992 to 2002 in the US, intentional drug poisoning constituted 7.1% of male suicides and 31% female suicides [5, 6]. Each method has its own challenges, and methods that are easily available (pesticides in developing countries, rope, and firearms) are used more frequently [5].

Over the past decade, suicide rates in the US have been steadily increasing [7]. In 2012, suicide was the 10th leading cause of death in the US [7, 8]. Similarly, suicide in Virginia has been increasing and is now the 11th leading cause of death [9]. In Virginia, suicides are also now three times more common than homicides [9]. Furthermore, the population composition in Virginia closely resembles national demographics. As of 2013, Virginia has a population of 8, 260, and 405 persons and females account for 50.8% of the population. Caucasians account for 63.6%, African Americans 19.7%, Asians 6.1%, Hispanics 8.6%, and Native Americans 0.1% of the Virginia population [10]. The average age of Virginia residents is 37.5 years [9].

In response to the high incidence of suicides, recent attention has focused on suicide prevention efforts. US suicide prevention strategies include physician education, lethal means restriction, pharmacotherapy, gatekeeper education, and psychotherapy [11]. The success of these strategies has varied considerably. Physician education, lethal means restriction, and gatekeeper education have had the greatest impact on decreasing suicide rates in the US [11].

Improving physician skills to recognize and manage risk factors for suicide has been shown to reduce rates of suicidal ideations in patients [12–14]. Prevention of Suicide in Primary Care Elderly: Collaborative Trial (PROSPECT) found that primary care intervention reduced suicidal ideations [12–14]. Studies have found that seventy percent of elderly suicide victims who committed suicide saw a primary care physician within a month of their death [12–14]. These data underscore the potential impact of primary care based screening and intervention strategies.

In addition to experienced clinicians and mental health care providers, laypersons with appropriate training can also be critical in helping to prevent suicides. Gatekeepers are typically identified as individuals who are in a position to recognize someone at increased suicide risk and may include clergy, first responders, and those employed in institutional settings such as the military [11]. Review of programs providing education and training in suicide prevention strategies to these gatekeepers has demonstrated positive effects. For example, implementation of the US Air Force suicide prevention program was associated with significant declines in suicide [15].

Policy-based strategies such as lethal means restriction have also yielded positive results. For example, limiting access to suicide methods such as CO has resulted in decreases in suicide by CO [16]. Restriction of other suicide methods has also shown positive results. For example, the implementation of enhanced restrictions to purchase firearms in the District of Columbia leads to reductions in firearm related suicides [17]. Implementation of means restriction is broad and can include (1) complete removal of a lethal method, (2) reducing the toxicity of a lethal method, for example, reducing CO content emissions from vehicles, (3) interfering with physical access, for example, using gun locks, (4) enhancing safety, for example, encouraging at risk families to remove lethal suicide means from the home, or (5) reducing the appeal of a more lethal method, for example, changing the perception of hanging as a quick and painless death [16, 18]. The majority of suicide attempts are transient and the time between contemplating suicide and the attempt is less than 5 minutes for many attempters [19]. Additionally, the method depends on what is readily available and certain methods such as firearms have a higher case fatality ratio [20]. Therefore restricting access to more lethal methods should theoretically save lives.

The goal of the current study is to examine and compare suicide rates and trends in Virginia from 2003 to 2012 specifically examining the method of suicide, the type of firearm, or mechanism of asphyxia used to commit suicide and demographics (age, gender, and race) to national trends. We will also provide healthcare practitioners with risk factors for completed suicide and inform policy makers of future directions for suicide prevention programs by reviewing these suicide trends. Existing Virginia preventive resources to help curb suicide will also be discussed.

## 2. Methods

We received IRB exemption for the study. Suicide data by method, gender, age, and race was extracted from Virginia's Office of the Chief Medical Examiners (OCME) annual report between 2003 and 2012. At the time of the study, only data from 2003 to 2012 was available.

Pursuant to § 32.1-283 of the Code of Virginia, the OCME has legal authority to investigate and accept deaths from trauma, injury, or violence, when sudden and unexpected, while unattended by a physician, under suspicious circumstances or in the custody of law enforcement or other state or local authority such as mental health facilities. The death must have occurred within the state of Virginia to become an OCME case. Each decedent's case information is entered into the Virginia Medical Examiner Data System (VMEDS), which allows for reliable case data. The Virginia OCME classifies these deaths by its own coding schema. Medical examiners use autopsy reports, police reports, hospital records, physician records, toxicology results, death investigations, and death scene reports to determine the cause of death. After taking into account all records and circumstances involving the death, the medical examiner certifies the cause and manner of death, which is reflected on the decedent's death certificate. Some suicides involve multiple methods but the medical examiners use their best judgment to determine the method that was responsible for the cause of death. While the OCME does not manage and certify all deaths reported in Virginia, all violent deaths, which includes all acts of suicides, fall under OCME jurisdiction, which allows for precise and very reliable data to be collected and analyzed. Additionally, the OCME oversees several public health surveillance projects and fatality review teams including the Virginia Violent Death Reporting System (VVDRS), which also collects, analyzes, and disseminates information about deaths due to violence, including suicide [21].

### 2.1. Suicide Method

Data is available on the method of suicide under the following categories: asphyxia, ingested and/or injected illicit or prescription drugs, burns, jumping from heights, CO poisoning, ingested and/or injected other types of poison such as ethylene glycol, stabbed self, firearm use, and vehicular and unknown traumatic causes.

Asphyxia was further subdivided into drowning, hanging, suffocation, strangulation, helium, plastic bag, oxygen replacement, other asphyxia and mechanical asphyxia. However due to changes in coding, strangulation and suffocation were replaced with other asphyxiation categories as a cause of death in 2006 and 2007, respectively. Firearm use was further subdivided into handgun, rifle, shotgun, and unspecified gun. A spearman's rho correlation was performed examining the overall change in the percentage of each method of suicide, method of asphyxia, and type of firearm used to commit suicide from 2003 to 2012.

### 2.2. Gender and Age

Suicide percentages by gender and age were divided into 10–14, 15–19, 20–24, 25–34, 35–44, 45–54, 55–64, 65–74, 75–84, and 85+ years for male and females. A spearman's rho correlation was performed examining the overall change in suicide for each age group by gender from 2003 to 2012.

### 2.3. Race

Suicide percentages by race were available under the following categories: Caucasians, African Americans, Hispanics, Asians, South East Asians, Native Americans, other, and unknown. However, since 2008, South East Asians have been grouped under the Asian category.

All statistical analyses were calculated using IBM SPSS software [30]. Significance was set at two-tailed *P* ≤ 0.05.

## 3. Results

Crude suicide rates in Virginia increased between 2003 and 2012 from 10.9/100,000 people to 12.9/100,00 people [21] (Table 1).

### 3.1. Relationship between the Method of Suicide and Year from 2003 to 2012

The most common method of suicide was firearm use, asphyxia, and ingested and/or injected illicit or prescription drugs, respectively (Table 1).

There was a significant increase in the percentage of asphyxiations (*r* = 0.77, *P* ≤ 0.01) as well as a significant decrease in the percentage of CO poisoning (*r* = −0.89, *P* < 0.01) and unknown traumatic causes (*r* = −0.90, *P* ≤ 0.01). The percentage of ingested and/or injected illicit or prescription drugs had a slight trend towards a significant decrease (*r* = −0.55, *P* = 0.1). There were no significant changes in burns (*r* = 0.31, *P* = 0.42), jumping from heights (*r* = 0.20, *P* = 0.58), ingested and/or injected other types of poison such as ethylene glycol (*r* = 0.36, *P* = 0.55), stabbing self (*r* = −0.17, *P* = 0.64), firearm use (*r* = −0.26, *P* = 0.47), and vehicular suicides (*r* = 0.52, *P* = 0.12).

### 3.2. Relationship between the Type of Firearm and Year from 2003 to 2012

**Figure 1:**
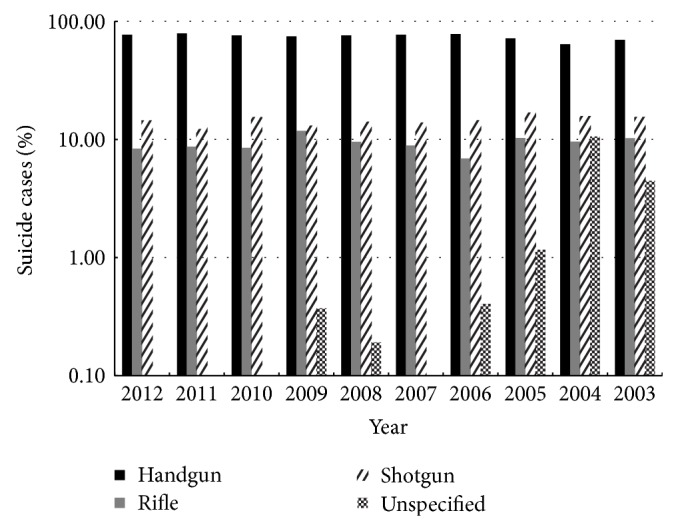
Mechanism of firearm.

The most common firearm used was handgun, shotgun, rifle, and unspecified weapon, respectively (Figure 1).

There was a significant decrease in unspecified weapons (*r* = −0.89, *P* = 0.02) and shotguns (*r* = −0.66, *P* = 0.04). The increase in handguns trended towards significance (*r* = 0.61, *P* = 0.06). There were no significant changes in the use of rifles (*r* = −0.47, *P* = 0.17).

### 3.3. Relationship between the Mechanism of Asphyxia and Year from 2003 to 2012

**Figure 2:**
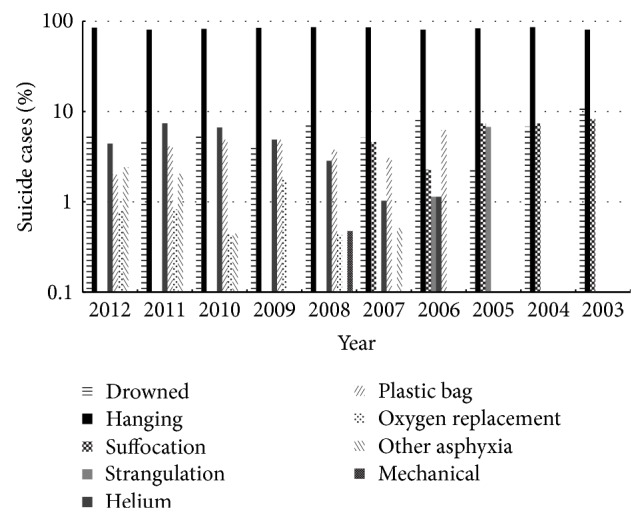
Mechanism of asphyxia.

The most common method of asphyxia was hanging (Figure 2).

The increase in helium (*r* = 0.75, *P* = 0.05), decrease in suffocation (*r* = −0.90, *P* = 0.04) and strangulation (*r* = −1.00, *P* ≤ 0.01) were significant. There were no significant changes in drowning (*r* = −0.37, *P* = 0.29), hanging (*r* = −0.02, *P* = 0.96), plastic bag (*r* = −0.41, *P* = 0.36), oxygen replacement (*r* = 0.21, *P* = 0.74), or other asphyxia (*r* = 0.80, *P* = 0.20).

### 3.4. Relationship between the Percentages of Male Suicide by Age Group from 2003 to 2012

The highest suicide percentage was in the 45–54-year-old age range (Table 2).

There was a significant increase in the percentage of male suicides in the 55–64-year-old age group (*r* = 0.79, *P* ≤ 0.01) as well as a significant decrease in the 10–14-year-old age group (*r* = −0.73, *P* = 0.02). The percentage of suicide in the 35–44-year-old age group had a trend towards a significant decrease (*r* = −0.60, *P* = 0.07). There were no significant changes in the percentages of male suicides in the age groups of 15–19 (*r* = −0.28, *P* = 0.43), 20–24 (*r* = −0.09, *P* = 0.80), 25–34 (*r* = 0.07, *P* = 0.86), 45–54 (*r* = 0.36, *P* = 0.31), 65–74 (*r* = −0.22, *P* = 0.53), 74–84 (*r* = −0.18, *P* = 0.96), and 85+ (*r* = 0.28, *P* = 0.43) years old.

### 3.5. Relationship between the Percentages of Female Suicide by Age Group from 2003 to 2012

The highest suicide percentage was in the 45–54-year-old age group (Table 2).

There was no significant changes in the percentages of female suicides in the age groups of 10–14 (*r* = 0.04, *P* = 0.91), 15–19 (*r* = 0.06, *P* = 0.88), 20–24 (*r* = 0.41, *P* = 0.24), 25–34 (*r* = −0.58, *P* = 0.08), 35–44 (*r* = −0.43, *P* = 0.21), 45–54 (*r* = 0.24, *P* = 0.51), 55–64 (*r* = 0.12, *P* = 0.75), 65–74 (*r* = 0.24, *P* = 0.51), 75–84 (*r* = −0.55, *P* = 0.1), and 85+ (*r* = −0.42, *P* = 0.23) years old.

### 3.6. Relationship between Race and Suicide from 2003 to 2012

**Figure 3:**
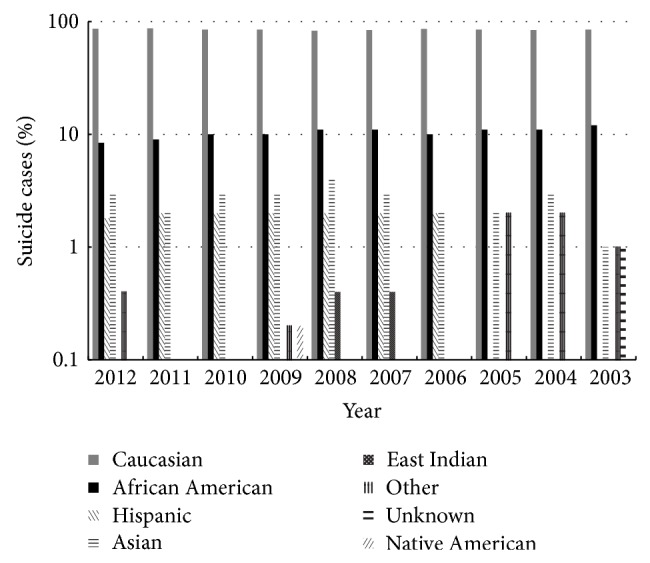
Percentage of suicide deaths by race from 2003 to 2012.

Suicide percentages by race remained mostly stable (Figure 3). Between 2003 and 2012, Caucasians represented the highest percentage of completed suicide followed by Africans Americans.

## 4. Discussion

The current study provides an analysis of Virginia suicide mortality data. Similar to Virginia, from 2003 to 2012, suicide rates in the USA increased from 10.8/100,000 to 12.6/100,000 people [7]. Firearms constitute the majority of suicides. The incidence of suicides by asphyxiations is increasing steadily. The incidence of suicide is highest in middle-aged adult females and males and increasing in the male 55–64-year-old age group. Completed suicide in Caucasians is higher than other race groups. However unlike national trends, intentional drug overdose deaths and male suicide in the 35–44-year-old age group and 10–14-year-old age group decreased. Furthermore, suicide in middle-aged females remained mostly stable.

### 4.1. Suicide Method

The Virginia data is consistent with national suicide trends. In both the US and Virginia, the most common suicide methods are firearm, asphyxia, and intentional drug overdose, respectively (see Table 1) [31].

#### 4.1.1. Firearms

Similar to Virginia, in the US, handguns constitute the majority of suicide by firearm and from 2003 to 2010, handgun suicides increased from 3,672 to 4,603 [32]. Kellermann and colleagues found that ready availability of firearms in the household was associated with an increased risk of suicide [33]. Studies have shown that the suicide risk is lower when firearms are stored securely, locked, and unloaded [34]. Based on these observations, the most effective method to reduce suicide would be to reduce access to firearms especially handguns. In Virginia, the Virginia Firearms Transaction Program and the Virginia State Police regulate firearm purchase (see Table 3) [27, 28]. More restrictive measures to purchase firearms especially handguns may reduce suicide rates. For example, the Virginia Firearms purchase eligibility test does not take into account a history of suicidal ideations or any prior suicide attempts. Adding a brief mental health evaluation to the firearm purchase eligibility test may help identify those at an increased risk of suicide. Widespread means restriction of firearms would theoretically have the greatest impact on reducing suicide rates but restricting gun access has been controversial in the US [16]. Other less intrusive ways to reduce access to firearms could be to have family members temporarily keep firearms away from immediate family members in an acute mental health crisis [16]. Due to the transient and impulsive nature of many suicide attempts, improvements in gun safety could also reduce suicide [18].

#### 4.1.2. Asphyxiations

Similarly in the US, from 2000 to 2010, asphyxiations increased by 52% [31]. The high prevalence of asphyxiations can be attributed to easy accessibility of rope and widespread availability of other means for hanging. Currently, there are no specific formal proposals on how to reduce asphyxiation suicides. More research is needed to develop interventions to curb the rise in asphyxiations. The easy availability of ligature materials makes prevention of hanging suicides a difficult task. Biddle and colleagues found that those who attempted suicide by hanging viewed it as a quick, simple, and painless death [35]. Therefore, one way to reduce hanging suicides would be to challenge perceptions of hanging as a quick, simple, and painless suicide method.

Increases in inert gas asphyxiations such as helium have also increased in the US and Virginia [36]. The increasing familiarity and lethality with helium is partly the reason for the rise in suicide by helium [36]. A guide called “Final Exit” gives directions on how to commit suicide with a plastic bag and helium [37, 38]. Additionally, there are a many internet websites that provide details on helium asphyxiations [39]. Furthermore, helium suicides have been publicized as simple and painless [37]. Helium suicides have also been on the rise in other countries [40]. More formal recommendations regarding suicides with inert gas asphyxiations such helium need to be developed. Besides physically restricting access to helium, one way to curb helium suicides would be to have professionals assess if at risk patients have read materials on helium suicides [36].

From 1999 to 2010, suicide by suffocation in the US increased from 18% to 24% for men and from 12% to 18% for women aged 35–64 years [41]. However, the decrease in strangulation and suffocation in Virginia can be attributed to changes in the collection and coding of data in 2006 and 2007, respectively. There is no data available for strangulation and suffocation suicides after 2006 and 2007, respectively, as both were subdivided into other asphyxia categories.

#### 4.1.3. Carbon Monoxide

Carbon monoxide suicides decreased in Virginia and since 1975, US intentional motor vehicle CO poisoning declined from 10.0 to 4.9 deaths per 1 million person-years [42]. This decrease can be attributed to stricter regulations limiting access to CO. In the past, suicide by CO poisoning was achieved by running a car's engine in a closed space. Since the US introduced catalytic converters in 1975 to reduce the amount of CO emitted by automobiles and the clean air act in 1970, there have been declines in CO poisoning [42]. Furthermore, Virginia has strict regulations regarding carbon monoxide detectors in residential buildings and prohibits tenants from tampering with carbon monoxide detectors [43]. Limiting access to CO has reduced CO suicides and is a prototypical model of the widespread success of lethal means restriction. Similar decreases in intentional CO poisoning were seen in the United Kingdom after the percentage of CO in domestic gas decreased from 13% in 1955 to 0% in 1975 [44].

#### 4.1.4. Other Suicide Methods

The decrease in unknown traumatic causes can be attributed to improved data collection and identification of the specific method of suicide.

In Virginia, there was a decrease in intentional drug overdoses; however in 2010, 17.20% of all US suicides were related to poisoning and from 2000 to 2010, US intentional poisoning increased from 1.7 per 100,000 people to 2.1 per 100,000 people [31, 45]. Expansion of Virginia's Drug Prescription Monitoring Program (PMP) may partially explain why there have been almost significant decreases in drug related suicides (see Table 3) [29]. The reliability and speed of prescription data collected by various Prescription Drug Monitoring Programs differs between states and studies have had mixed findings on the impact of PMPs on overall drug use [46–48]. In comparison to other states, Virginia is part of the Prescription Monitoring Program Interconnect (PMPi), which allows PMP users to effectively see a more complete history of patients controlled substance use in other member states (see Table 3) [29]. Overall, PMPs are important to prevent nonmedical use of prescribed controlled substances and help reduce diversion and fraudulent prescribing [49]. Other states with high rates of intentional prescription overdose related deaths should consider implementing a PMP.

### 4.2. Suicide by Age and Gender

In both the US and Virginia, males commit suicide more so than females. The majority of suicides in males and females occur during the middle age (see Table 2).

#### 4.2.1. Middle Age (35–64 Years)

From 1999 to 2010, the suicide rate for adults in the US aged 35–64 years increased significantly by 27.3%; however for all other age groups (10–34 years and ≥65 years), there were no statistically significant changes in suicide rates [41]. One reason for the national increase in suicide in middle-aged adults has been the economic recession [50]. Unemployment causes a decline in mental health, increasing the risk for suicide [51, 52]. However, Virginia's unemployment rate has remained much lower than the national average at 5.6% in 2013 and has been declining since 2009, which may explain the decrease in middle-aged male suicides (35–44-year-old age group) and relatively stable suicide percentages in females. In Virginia, the increase in male suicides in the 55–64-year-old age group may be attributable to other later life stressors such as loss, medical illness, and functional status [53]. The increase in male suicides in the 55–64-year-old age group may also be explained by a birth cohort effect [54]. Cohorts born between 1945 and 1964 have had higher suicide rates in their teenage years and thus the increase may be due the higher suicide rate of cohorts entering the later middle age period [54]. In response to increased suicide in the later middle ages, the American Foundation for suicide prevention has recently developed a training course in Virginia to help clinicians recognize clients at risk for suicide (see Table 3) [24]. Physician education in depression recognition would reduce suicide rates in all age groups especially in elder middle-aged adults who are more likely to see a primary care provider [11].

#### 4.2.2. Other Age Groups

Similarly to national trends in the 65+ age group, it is encouraging to see that there were no significant increases in suicide [41]. However, in contrast to national trends, Virginia experienced a significant decrease in suicide in males aged 10–14 years [41]. The decrease in adolescent male suicides in Virginia has been associated with implementation of the Suicide Prevention Guidelines for Virginia by the Virginia State Board of Education in 2003 [22] (see Table 3). Similarly, other studies have shown that youth specific targeted suicide prevention programs have resulted in decreases in youth suicidal behaviors [55].

### 4.3. Suicide by Race

In the US, from 2005 to 2009, the highest suicide rate for men older than 10 years of age occurred in American Indian/Alaskan Native males at 27.61 suicides per 100,000 people followed by Non-Hispanic White males at 25.96 suicides per 100,00 people [56]. Non-Hispanic African American males had a suicide rate of roughly 11 suicides per 100,000 people [56]. The highest suicide rate for females older than 10 years of age occurred in American Indian/Alaskan Natives at 7.87 per 100,000 people followed by Non-Hispanic Whites at 6.71 per 100,000 people [56]. Non-Hispanic African American females had a suicide rate of roughly 2 suicides per 100,000 people [56]. The higher percentage of Caucasians committing suicide in Virginia can be partially attributed to population demographics. In 2012, in Virginia, Caucasians comprised 71.1%, African-Americans 19.7%, Hispanics 8.4%, Asians 6.0%, and Native Americans 0.5% of the population [57]. However, a study found that lower suicide rates in African-Americans compared to Caucasians could be explained by protective factors in African-Americans [58]. However, the participants were relatively high socioeconomic college students, limiting its generalizability [58].

Although Asians and Hispanics comprised only 8.4% and 6.0% of Virginia, respectively, each still accounted for 3% and 1.8% of suicide, respectively, in 2012. Female gender, family conflict, perceived discrimination, and the presence of a depressive or anxiety disorder are risk factors for suicide in Asian Americans [59]. A high level of identification with one's ethnic group was a protective factor for Asian Americans [59]. A lifetime DSM-IV diagnoses, female gender, acculturation, and high levels of family conflict are suicide risk factors for Latinos in the US [60]. As the US population becomes increasingly more diverse, more specific suicide preventative resources will need to be targeted towards ethnic minorities.

### 4.4. Virginia Suicide Prevention Programs

The main strategies of Virginia suicide prevention are to (1) improve and expand suicide surveillance systems, (2) develop community based suicide prevention programs, (3) reduce access to lethal means, (4) implement training to recognize at risk patients, (5) promote suicide awareness, and (6) increase access to mental health services [61]. A review of suicide prevention programs is provided in Table 3 including suicide prevention programs implemented by the Virginia Department of Health and the Virginia Association of Community Services Boards [23, 25, 26]. Furthermore, the Virginia Community Services Boards provide comprehensive mental health crisis care, which has been associated with the largest reduction in suicide rates in all age groups (see Table 3) [62].

## 5. Conclusion

There have been increases in suicide in the US, including Virginia. Overall, suicide rates and trends in Virginia closely resemble national rates albeit some differences. Suicide by firearm and asphyxiation remains a significant cause of suicide mortality. Due to similarities between US suicides and Virginia suicides, future successful national suicide prevention policies should also be implemented on a smaller scale in Virginia. Although implementation of specific suicide preventive resources in Virginia (see Table 3) may have helped to reduce overall suicide mortality, further preventive measures are needed to reduce escalating suicide rates. Decreases in suicide have been associated with increased surveillance such as implementation of the Virginia PMP as well as age specific interventions such as implementation of suicide prevention guidelines by the Virginia State Board of Education (see Table 3). Lethal means restriction of CO can be used as model to reduce other suicide methods such as firearms, inert gases, and asphyxiations. Stricter monitoring of narcotic prescriptions may help reduce intentional drug overdoses. Suicide policies directed towards high-risk groups such as middle-aged adults, Caucasians, and an increasingly diverse population could help reduce suicide rates. Gatekeepers and mental health care workers should be cognizant of risk factors for completed suicide and incorporate these into suicide risk assessments.

Overall, there have been significant improvements in our understanding of risk factors for suicide but future research should focus on the development of empirically based suicide prevention programs and protocols.

## Figures and Tables

**Table 1:** Suicide rate and method in Virginia from 2003 to 2012.

Year	Total suicide cases (*n*)	Suicide rate per 100,000 people	Asphyxia (%)	Ingested and/or injected illicit or prescription drugs (%)	Burns (%)	Jumping from heights (%)	CO poisoning (%)	Ingested and/or injected other types of poison such as ethylene glycol (%)	Stabbed self (%)	Firearm use (%)	Unknown traumatic causes (%)	Vehicular (%)
2012	1,053	12.9	23.65	13.20	0.47	1.80	1.71	0.95	1.04	55.56	0.10	1.52
2011	1067	13.2	22.76	12.93	Not available	2.25	1.59	0.94	1.31	57.08	0.09	1.03
2010	996	12.4	22.58	11.95	0.90	2.11	1.71	0.30	1.31	57.73	Not available	1.31
2009	974	12.4	23.01	14.68	0.41	1.64	1.85	Not available	1.74	55.34	0.10	1.13
2008	949	12.2	22.23	14.33	0.11	2.11	2.00	0.95	2.32	55.11	0.11	0.74
2007	906	11.7	20.53	16.56	0.44	1.77	3.09	0.55	1.55	54.53	0.22	0.77
2006	884	11.6	19.91	15.72	0.34	2.72	2.49	Not available	1.81	55.77	0.11	1.13
2005	875	11.6	18.63	13.26	0.34	2.4	2.51	Not available	1.94	58.74	1.02	1.14
2004	840	11.3	20.95	13.33	0.48	1.19	2.50	Not available	1.43	59.52	0.24	0.36
2003	804	10.9	21.13	14.93	0.37	1.74	3.73	Not available	0.75	55.72	0.62	0.99

**Table 2:** Suicide for males and females by age range in Virginia from 2003 to 2012.

Year	10–14	15–19	20–24	25–34	35–44	45–54	55–64	65–74	75–84	85+	Unknown age
	(%)	(%)	(%)	(%)	(%)	(%)	(%)	(%)	(%)	(%)	(%)
Males											
2012	0.37	4.15	7.93	16.95	16.71	19.02	16.71	7.56	7.56	3.05	Not available
2011	0.24	4.95	8.45	15.34	13.77	23.07	15.22	9.18	7.25	2.54	Not available
2010	0.51	4.23	6.79	16.92	16.15	23.46	14.62	8.97	5.00	3.33	Not available
2009	0.13	4.50	7.55	13.91	19.74	22.38	17.09	8.34	4.11	2.25	Not available
2008	0.81	5.54	8.65	14.46	16.62	22.84	12.70	9.59	6.89	1.89	Not available
2007	0.60	4.17	9.24	12.82	20.57	20.12	14.46	7.00	8.49	2.53	Not available
2006	0.45	4.17	10.13	13.86	18.03	22.65	13.71	8.49	5.96	2.53	Not available
2005	1.15	3.46	7.22	17.89	17.75	21.07	14.29	8.37	6.35	2.45	Not available
2004	0.60	5.11	7.66	13.81	20.42	19.67	13.06	8.41	7.96	3.15	0.15
2003	1.13	5.65	8.06	17.74	18.87	19.19	9.68	10.48	6.29	2.42	0.48

Females											
2012	0.86	3.43	6.44	14.16	21.03	26.61	16.74	6.87	3.0	0.86	Not available
2011	0.84	2.93	9.21	12.55	18.83	24.69	17.99	10.46	1.67	0.84	Not available
2010	0.00	3.70	4.63	13.89	23.61	27.78	15.28	4.63	4.17	2.31	Not available
2009	0.46	3.20	4.57	11.42	25.11	26.94	19.63	5.94	1.83	0.91	Not available
2008	0.96	4.31	5.74	16.27	24.88	19.14	19.62	3.35	2.87	2.87	Not available
2007	0.00	2.98	3.40	16.17	22.98	31.06	13.19	6.38	2.55	1.28	Not available
2006	0.00	2.82	4.69	13.62	21.13	28.64	15.49	6.57	6.10	0.94	Not available
2005	2.20	6.59	5.49	14.29	18.68	23.63	19.78	4.94	3.30	1.10	Not available
2004	0.57	4.02	5.75	15.52	28.16	26.44	11.49	2.87	4.02	1.15	Not available
2003	0.54	1.09	4.35	16.30	26.09	20.11	16.85	7.07	5.98	1.63	Not available

**Table 3:** Suicide preventive resources.

Suicide reduction programs	
2003 Suicide Prevention Guidelines for Virginia by the Virginia State Board of Education [22].	Provides guidelines for school personnel when they suspect a student is at risk for suicide: (i) Responding to the suicidal child (ii) Characteristics to identify potentially suicidal students. (iii) Criteria to assess the suicide risks of students (iv) Suicide prevention strategies (v) Imminent suicide warning signs

2010 Virginia Suicide Prevention Resources Directory [23].	The directory provides a list of national, state, and local resources that are available when people are impacted by suicide: (i) Community mental health centers (ii) Crisis hotlines (iii) Youth hotlines (iv) Statewide mental health facilities (v) Coalitions to educate the public in suicide prevention and intervention (vi) Survivors of suicide loss support groups (vii) Veterans services (viii) Local suicide prevention websites (ix) Local mental health centers

Suicide Prevention Resource Center and the American Foundation for Suicide Prevention [24].	Provides an essential skills training course to help clinicians recognize clients at risk for suicide. There are several components to this course including two online courses, self-paced modules, and a two-day face-to-face workshop. The Virginia Department of Health has recognized the importance in clinician recognition of depression in suicide prevention. In 2013, the Virginia Department of Health held three different two-day training sessions to help clinicians recognize and respond to depression.

Virginia Association of Community Services Boards (VACSB) [25].	The Community Services Boards (CSBs) provide publicly funded services for mental health, intellectual disability, and substance abuse. Community service boards provide preadmission screening services 24 hours per day, 7 days per week. In Virginia, there are 39 CSBs that provide mental health crisis care. To improve the quality of services, VACSB hosts one public policy and one legislative and one professional development conference each year.

Virginia Department of Health [26].	QPR (Question, Persuade, Refer): 1-2-hour gateway training for the public to bring awareness to suicide and inform community members about warning signs for suicidal behaviors. SafeTALK (Suicide Alertness for Everyone; Tell, Ask, Listen and Keep Safe): Teach participants to recognize suicidal persons and connect them with suicide intervention community resources. ASIST (Applied Suicide Intervention Skills Training): Two-day course to help caregivers recognize risk for suicide and intervene to prevent immediate harm. RRSR (Recognizing and Responding to Suicide Risk: Essential Skills for Mental Health Clinicians): 2-day training course to establish core competencies that mental health professionals need in order to manage suicide risk RRSR-PC (Recognizing and Responding to Suicide Risk: Essential Skills for Primary Care Providers): Provides 90-minute training on suicide risk assessment to physicians, nurses/nurse practitioners, and physician assistants

Firearm regulation	

1989 Virginia Firearms Transaction Program (VFTP) [27].	(i) Regulates firearm purchase (ii) Decision regarding the sale of all firearms based upon criminal history record information (CHRI) (iii) Firearm purchaser is scanned in 5 different national and state databases: (1) Virginia's wanted and missing persons files and protective orders (2) Virginia's criminal history record files (3) Virginia's database of adjudications of legal incompetence and incapacity (4) Involuntary commitments to mental health institutions for inpatient or outpatient treatment (5) National Instant Criminal Background Check System (iv) Provides further regulations and guidelines for the sellers of firearms

2009 Virginia State Police [28].	Provides an eligibility test for firearm purchase. A history of violence or (since 2009) involuntary admission to a psychiatric facility may prohibit ownership of a firearm. Provisionally, the firearm purchase test does not directly take into account a history of suicidal ideations or any prior suicide attempts. The test is available online at http://www.vsp.state.va.us/Firearms_PurchaseEligibility.shtm ^***^Adding a brief, concise, mental health evaluation to the firearm purchase eligibility test may help identify those at an increased risk of suicide

Narcotic regulation	

Virginia's Online Prescription Monitoring Program (PMP) [29].	The PMP provides a database history of schedule II–IV prescriptions with a mission of promoting appropriate use of controlled substances. Registered users can access the database and determine if a patient is abusing controlled substances. As of 2011, Virginia is part of the National Association of Boards of Pharmacy Prescription Monitoring Program InterConnect (PMPi), which comprises several states, which share prescription drug information.

## Limitations

The study was descriptive in design, and the complex relationship between suicide risk factors was beyond the scope of this study. Suicides may be slightly underreported in Virginia as the burden of proof should exceed “more likely than not” to classify a death as suicide.
